# Doctors should choose communication strategies based on the patient's attitude toward disease and healthcare workers: a study in Jiangsu, China

**DOI:** 10.3389/frhs.2025.1520628

**Published:** 2025-07-03

**Authors:** Fangjing Zheng, Aicui Lin

**Affiliations:** ^1^ Department of Thoracic and Cardiovascular Surgery, Nanjing First Hospital, Nanjing Medical University, Jiangsu, China; ^2^ Department of Science and Technology, Nanjing First Hospital, Nanjing Medical University, Jiangsu, China

**Keywords:** communication, doctor-patient relationship, education level, economy, higher education

## Abstract

**Objective:**

We aimed to determine patients’ expectations regarding curing their disease and potential communication preferences with healthcare providers, by analyzing factors such as education level, age, type of medical visit, and residential region, so as to assist healthcare workers in managing communication more effectively.

**Methods:**

A sampling survey was conducted involving 1,155 patients across nine public tertiary hospitals in Jiangsu Province, China. The survey questionnaire results were verified and organized, after which chi-square tests and *Z*-tests were conducted to analyze the responses to each question across different groups.

**Results:**

Among the educated population, the proportion of patients who believe that diseases can definitely be cured tends to decrease as the level of educational increases. Similarly, this proportion also tends to decrease with an increase in economic development across different regions. The proportion of patients who believe that they should fulfill their obligations during the treatment process but lacked understanding of how to do so also exhibits a similar trend. Adults under the age of 50, as well as outpatient and emergency patients, are more willing to cooperate with medical treatment, but often lack knowledge about how to do so.

**Conclusion:**

Healthcare workers should select communication strategy that are suitable for patients considering their age, education, type of medical visit, and residential region and provide appropriate cure expectations to prevent communication issues.

## Highlights

• Education and economic levels may affect patients' expectation of disease.
• Education and regional economic levels affect patients' communication mode.
• Patients in outpatient/emergency departments wish to better cooperate with staff.
• Medical staff should adjust communication strategies according to patient characteristics.

## 1 Background

The doctor–patient relationship is at the core of interpersonal interactions in the medical environment (1). A good doctor–patient relationship is beneficial to patient recovery (2), whereas a hostile one can affect loyalty and potentially lead to medical disputes. The rising trend of doctor–patient conflicts in China was previously reported (3) and has led to a focus on the relationship and communication.

Studies have shown that improved communication skills can enhance interactions between doctors and patients (4) and thereby prevent medical disputes. Moreover, patient-centered communication theories have been developed in recent decades (5, 6) and are considered crucial to improving medical care quality and outcomes (7). Patient-centered communication requires doctors to treat patients kindly, listen to patients' statements, and empathize with patients. However, considering the cultural and background differences in medical practice, this communication style may not be entirely applicable in all situations (8).

In China, public hospitals play a dominant role in healthcare work (9), especially with rapid aging of the population (10, 11) and urbanization (12, 13), leading to a large patient volume in public class A tertiary hospitals. To prevent dissatisfaction among large numbers of patients awaiting medical treatment, with doctors facing increasing pressure in diagnosis and treatment, they often must complete a patient's treatment within a short time, leaving insufficient time to listen to patients' statements, which usually lack professional clarity and contain many unnecessary details. When doctors need to provide effective medical services within a short time (2), communication strategy that require a lot of time become difficult to adopt in Chinese doctor–patient communication.

Poor communication is the main factor involved in medical disputes (14), and limited time is the fundamental reason for poor communication. This article suggests that, in China, the key to improving communication strategies is determining how medical staff can provide effective medical services and identify patients who are more likely to be dissatisfied so as to take appropriate measures and prevent the occurrence of patient dissatisfaction.

Cultural and educational backgrounds are important factors in medical disputes that affect patient communication preferences (15), yet few studies have addressed this. In the present study, we analyzed patients' cognitive levels and communication preferences regarding healthcare from different perspectives such as age, education level, type of medical visit, and residential region, aiming to identify patterns so as to help medical staff treat patients more effectively and reduce conflicts.

## 2 Participants and methods

### 2.1 Participants

Based on political and economic factors and the distribution of class A tertiary hospitals in Jiangsu Province, we used a stratified random sampling method to select one class A tertiary hospital each from Changzhou, Yangzhou, and Huai'an City as representative of Southern, Central, and Northern Jiangsu; six class A tertiary hospitals belonging to two types (comprehensive hospitals and traditional Chinese medicine hospitals) in Nanjing were selected, totaling nine hospitals surveyed.

### 2.2 Methods

#### 2.2.1 Questionnaire design

The initial questionnaire was designed based on a literature search and was refined by experts in hospital management and medical humanities. After pre-survey testing and reliability and validity testing, the questionnaire was administered to participants.

#### 2.2.2 Survey and quality control

Before the survey, the purpose, importance, and completion instructions were explained to respondents. Respondents completed the questionnaire independently. For illiterate respondents, the questionnaire was read to them by surveyor administrators who marked each choice according to the respondent's selection. The questionnaire data were reviewed for completeness and logical accuracy, with questionnaires missing more than 10% of items considered invalid. A total of 1,155 questionnaires were distributed, with 1,088 valid responses, an effective recovery rate of 94.2%.

### 2.3 Statistical methods

EpiData software version 3.1 (Odense, Denmark) was used for double entry and verification of questionnaires and database establishment. We used SPSS version 13.0 (SPSS Inc., Chicago, IL, USA) for statistical analysis. Chi-squared tests and *Z*-tests were used to analyze the responses to each question across different groups. *P* < 0.05 was considered statistically significant.

## 3 Results

### 3.1 Patients' perspectives on treatment effectiveness

The demographic characteristics of the respondents are shown in Table 1. When asked, “What do you think is the most reasonable option regarding the treatment effect of a disease?” there were certain differences in patients' choices under different situations. The proportion of patients who believed that diseases can definitely be cured exhibited an inverse U-shaped relationship with educational level, and this trend was statistically significant (Table 2, *P* < 0.05). There was no difference among patients in different age groups or among those with different types of medical visits (*P* > 0.05). In terms of residential region, the proportion of those from the Northern Jiangsu region who believe that diseases can definitely be cured was significantly higher than that of other regions (*P* < 0.05).

**Table 1:** Demographic characteristics of respondents.

Category	Group	Number	Percentage
Age	<19 years	143	13.14%
	19–30 years	203	18.66%
	31–40 years	185	17.00%
	41–50 years	330	30.33%
	≥51 years	227	20.86%
Education	Illiterate	73	6.71%
	Primary school	121	11.12%
	Junior high school	194	17.83%
	High school	157	14.43%
	College degree	227	20.86%
	Bachelor's degree or above	306	28.13%
	Null	10	0.92%
Region	Nanjing	524	48.16%
	Southern Jiangsu	180	16.54%
	Central Jiangsu	154	14.15%
	Northern Jiangsu	216	19.85%
	Null	14	1.29%

**Table 2:** Perception of treatment effectiveness.

Category	Group	Misdiagnosis and mistreatment are inevitable	Many diseases cannot be cured	All diseases can be cured	Total	*χ* ^2^	*P*
Education	Illiterate	23 (31.5%)	30 (41.1%)	20 (27.4%)	73 (100%)	27.610	0.002
	Primary school	23 (19.0%)	45 (37.2%)	53 (43.8%)	121 (100%)		
	Junior high school	44 (23.0%)	59 (30.9%)	88 (46.1%)	191 (100%)		
	High school	44 (28.6%)	50 (32.5%)	60 (39.0%)	154 (100%)		
	College degree	58 (25.8%)	79 (35.1%)	88 (39.1%)	225 (100%)		
	Bachelor's degree or above	101 (33.2%)	119 (39.1%)	84 (27.6%)	304 (100%)		
Age	<19 years	60 (26.5%)	89 (39.4%)	77 (34.1%)	226 (100%)	5.643	0.687
	19–30 years	36 (25.4%)	54 (38.0%)	52 (36.6%)	142 (100%)		
	31–40 years	59 (29.4%)	69 (34.3%)	73 (36.3%)	201 (100%)		
	41–50 years	56 (30.4%)	66 (35.9%)	62 (33.7%)	184 (100%)		
	≥51 years	84 (25.8%)	108 (33.2%)	133 (40.9%)	325 (100%)		
Visit Type	Outpatient services	115 (30.3%)	136 (35.9%)	128 (33.8%)	379 (100%)	8.126	0.087
	Hospitalization	148 (24.1%)	225 (36.6%)	241 (39.3%)	614 (100%)		
	Emergency department	26 (35.1%)	22 (29.7%)	26 (35.1%)	74 (100%)		
Region	Nanjing	151 (29.2%)	188 (36.4%)	178 (34.4%)	517 (100%)	15.552	0.016
	Southern Jiangsu	36 (20.0%)	78 (43.3%)	66 (36.7%)	180 (100%)		
	Central Jiangsu	50 (32.7%)	50 (32.7%)	53 (34.6%)	153 (100%)		
	Northern Jiangsu	54 (25.2%)	65 (30.4%)	95 (44.4%)	214 (100%)		

### 3.2 Patients' perspectives on obligations

Among patients with an associate's degree or above, over 50% believed that they should cooperate with doctors but often lacked knowledge about how to do so (Table 3, *P* < 0.05). Among adult patients under 50 years old, the proportion holding this view was higher than 46.5%, and this result was statistically significant (*P* < 0.05). The proportion of outpatient and emergency patients holding this view was higher than that of inpatients (*P* < 0.05). In terms of residential region, the proportion of patients holding this view was highest among patients in Nanjing (the capital of Jiangsu Province, located in the province's southern area), and then it decreased gradually from southern Jiangsu to northern Jiangsu, with significant differences between populations (*P* < 0.05).

**Table 3:** Self-evaluation during the diagnosis and treatment process.

Category	Group	Patients are a vulnerable group and should not have too many obligations	Patients should fulfill their obligations, but many patients lack an understanding of them	Patients understand their obligations and perform poorly in diagnosis and treatment	Patients understand their obligations and perform well in diagnosis and treatment	Total	χ^2^	*P*
Education	Illiterate	12 (16.7%)	26 (36.1%)	2 (2.8%)	32 (44.4%)	72 (100%)	66.802	0
	Primary school	32 (26.4%)	37 (30.6%)	10 (8.3%)	42 (34.7%)	121 (100%)		
	Junior high school	58 (30.2%)	65 (33.9%)	11 (5.7%)	58 (30.2%)	192 (100%)		
	High school	43 (27.4%)	51 (32.5%)	13 (8.2%)	50 (31.8%)	157 (100%)		
	College degree	33 (14.6%)	115 (50.9%)	11 (4.9%)	67 (29.6%)	226 (100%)		
	Bachelor's degree or above	36 (11.8%)	162 (53.3%)	10 (3.3%)	96 (31.7)	304 (100%)		
Age	<19 years	43 (19.0%)	83 (36.7%)	29 (12.8%)	71 (31.4%)	226 (100%)	66.076	0
	19–30 years	18 (12.7%)	66 (46.5%)	5 (3.5%)	53 (37.3%)	142 (100%)		
	31–40 years	29 (14.4%)	108 (53.5%)	8 (4.0%)	57 (28.2%)	202 (100%)		
	41–50 years	34 (18.4%)	91 (49.2%)	6 (3.2%)	54 (29.2%)	185 (100%)		
	≥51 years	91 (27.8%)	112 (34.3%)	10 (3.1%)	114 (34.9%)	327 (100%)		
Visit type	Outpatient services	75 (19.6%)	187 (49.0%)	19 (5.0%)	101 (26.4%)	382 (100%)	15.218	0.019
	Hospitalization	124 (20.1%)	232 (37.7%)	34 (5.5%)	226 (36.7%)	616 (100%)		
	Emergency department	14 (19.2%)	33 (45.2%)	4 (5.5%)	22 (30.1%)	73 (100%)		
Region	Nanjing	109 (20.9%)	244 (46.8%)	16 (3.1%)	152 (29.2%)	521 (100%)	48.813	0
	Southern Jiangsu	35 (19.6%)	74 (41.3%)	12 (6.7%)	58 (32.4%)	179 (100%)		
	Central Jiangsu	32 (20.9%)	59 (38.6%)	23 (15.0%)	39 (25.5%)	153 (100%)		
	Northern Jiangsu	38 (17.7%)	80 (37.2%)	7 (3.3%)	90 (41.9%)	215 (100%)		

## 4 Discussion

This research, based on a survey and analysis of patients visiting class A tertiary hospitals in Jiangsu Province, found that education level has a significant impact on patients, both in their expectations for curing their disease and their attitudes during doctor–patient communication. Specifically, the higher the education level, the greater the proportion of patients who demonstrate rational attitudes. This result is similar to Asjat's research (16) about patient's educational level and satisfaction with the quality of the mandibular denture bearing area and Doaa's research (17) about drug services. This work suggests that this phenomenon may be explained by the fact that patients with lower levels of literacy often hold overly optimistic expectations regarding curing their disease, while those with a bachelor's degree or higher tend to approach medical visits more rationally due to their higher health literacy. Saisai's research (18) also came to the same conclusion. The study also found that among illiterate patients, the proportion of those who believed their disease could definitely be cured was not high. Because patients' experiences and backgrounds may influence their communication with doctors (19), this article suggests that illiterate patients, who are often older individuals with rich life experience, tend to have more rational expectations regarding the cure of their diseases.

In Jiangsu Province, the gross enrollment rate for higher education reached 54.7% in 2016 (20) and 60.2% in 2021 (21). Patients with higher education know how to reflect on themselves and are thus able to better communicate with doctors (22). Therefore, a large proportion of patients aged 19–50 years believe that they lack understanding about how to cooperate with doctors.

In regions with a higher economic level and social development, patients achieve higher levels of education (23). Research has pointed out that the level of economic development in Jiangsu Province improves from north to south, and the proportion of highly educated individuals in Jiangsu also increases from north to south (24). Therefore, in southern Jiangsu, where the level of economic development is high and the proportion of highly educated individuals is large, the proportion of rational patients is also high.

Compared with inpatients, outpatient and emergency patients have little time to interact with medical staff (25, 26). As patients need to cooperate with medical staff to conduct various examinations and make decisions in a short period of time, it is difficult for them to do these things well with limited medical knowledge; thus, outpatient and emergency patients are more likely to realize their limitations in this regard. This explains why the proportion of outpatient and emergency patients choosing the second option, i.e., that patients should fulfill their obligations but that many patients lack an understanding of them, is significantly higher than those choosing any other option in Table 3.

Based on the above results, two suggestions can be made. First, medical staff in different regions should manage patients' expectations of being cured by taking into account the local area and the patients' education level when interacting with them. For example, medical staff should be more cautious about giving overly optimistic expectations of cures to patients with lower education levels and those from northern Jiangsu to prevent subsequent medical disputes if the optimistic prognosis is not achieved. Second, when diagnosing and treating patients, medical staff should choose the most suitable communication methods based on the patients' age, education level, type of medical visit, and residential origin. For example, when facing adult patients with high education levels in outpatient or emergency departments, explaining in detail how they need to cooperate with the diagnosis and treatment is more conducive to communication. For patients who are over 60 years old, have a lower level of education, or are from northern Jiangsu, it is recommended to show humanistic care through gentle words and tones. This is more conducive to building a harmonious doctor–patient relationship.

This study has some limitations. We only conducted a sampling survey of public class A tertiary hospitals in Jiangsu Province. Since Jiangsu's overall economic strength is among the highest in China, it is uncertain whether the suggestions are applicable to provinces with weaker economies or to other countries. Additionally, the survey was limited to public class A tertiary hospitals, which have a significantly different daily patient volume than primary and secondary hospitals; therefore, the suggestions may only be applicable to public class A tertiary hospitals.

## 5 Conclusions

This survey of patients in public class A tertiary hospitals in Jiangsu Province found that the higher the education level and the higher the economic development level of the region, the higher the proportion of rational patients seeking medical care. Additionally, outpatient and emergency patients are more willing to learn how to cooperate with doctors’ diagnosis and treatment than inpatients are. Thus, it is recommended that medical staff in different regions choose appropriate doctor–patient communication strategies.

## Data Availability

Inquiries about the original data presented in the study can be directed to the corresponding author.
